# Exogenous IFN-alpha Administration Reduces Influenza A Virus Replication in the Lower Respiratory Tract of Rhesus Macaques

**DOI:** 10.1371/journal.pone.0029255

**Published:** 2011-12-29

**Authors:** Shannon R. Matzinger, Timothy D. Carroll, Linda Fritts, Michael B. McChesney, Christopher J. Miller

**Affiliations:** 1 Center for Comparative Medicine, University of California Davis, Davis, California, United States of America; 2 California National Primate Research Center, University of California Davis, Davis, California, United States of America; The University of Hong Kong, China

## Abstract

To determine the role of innate immune responses in controlling influenza A virus replication, rhesus macaques (RM) were administered pegylated IFN-alpha prior to virus challenge. Systemic and mucosal pegylated IFN-alpha administration induced expression of the interferon-stimulated genes (ISG) MxA and OAS in the airways. RM treated with IFN-alpha 24 hours prior to influenza virus challenge had significantly lower peak vRNA levels in the trachea compared to untreated animals. In addition to blunting viral replication, IFN-alpha treatment minimized the weight loss and spike in body temperature after influenza infection of RM. These results confirm the importance of IFN-alpha induced innate immune responses in the rapid control of influenza A virus replication in primates.

## Introduction

Seasonal influenza is an acute respiratory tract disease of humans that is highly contagious and results in high morbidity and mortality each year, mainly among the young, elderly, and immune-compromised [1]. Influenza A virus replication peaks within the first few days of infection and is cleared 5–7 days after infection, well before the generation of virus-specific adaptive immune responses [2]. Thus, it is unlikely that the adaptive immune response plays an important role in clearing the virus. In contrast, the innate immune response, specifically the expression of type I interferons (IFN), is induced within hours of infection and the peak of IFN-alpha-induced gene expression temporally correlates with the decrease in viral replication in humans [3].

Type I IFNs induce the expression of hundreds of antiviral effector molecules [4], which have been shown to play important roles in blocking virus replication, though the specific interferon-stimulated genes involved varies with the virus [5]. Administering Type I IFN prior to influenza A virus inoculation reduces virus replication in the upper respiratory tract mice and ferrets [6], [7], [8], however, a role for IFN-alpha in controlling influenza virus replication in humans or the lower respiratory tract of mice and ferrets has never been demonstrated. Due to the relatively close phylogenetic relationship of nonhuman primates to humans, macaques are excellent models of the human immune and respiratory systems. Here we report that the administration of IFN-alpha to rhesus macaques prior to Influenza A virus inoculation significantly decreases clinical signs of infection and reduces virus replication in the lower respiratory tract.

## Methods

### Ethics Statement/Animals

All animals used in this study were adult rhesus macaques (Macaca mulatta) housed at the California National Primate Research Center in accordance with the recommendations of the Association for Assessment and Accreditation of Laboratory Animal Care International Standards and with the recommendations in the Guide for the Care and Use of Laboratory Animals of the National Institutes of Health. The Institutional Animal Use and Care Committee of the University of California, Davis, approved these experiments (Protocol #13022). For blood collection, animals were anesthetized with 10 mg/kg ketamine hydrochloride (Park-Davis) injected i.m. For virus inoculation and respiratory secretion sample collection, animals were additionally anesthetized with 15–30 µg/kg Domitor (Orion Pharma) injected i.m., and anesthesia was reversed with 0.07–0.15 mg/kg Antisedan (Pfizer Animal Health) injected i.m. All efforts were made to minimize suffering. Details of animal welfare and steps taken to ameliorate suffering were in accordance with the recommendations of the Weatherall report, “The use of non-human primates in research”. Animals were housed in an air-conditioned facility with an ambient temperature of 21–25°C, a relative humidity of 40%–60% and a 12 h light/dark cycle. Animals were individually housed in suspended stainless steel wire-bottomed cages and provided with a commercial primate diet. Fresh fruit was provided once daily and water was freely available at all times.

### Virus Stocks

The human influenza A virus isolate used in this study, A/Memphis/7/2001 (H1N1), was generously provide by Richard Webby at the St. Jude's Children Hospital, Memphis, TN. This isolate was isolated on MDCK and was not passaged further prior to expansion in MDCK cells (American Type Culture Collection, Manassas, VA) to produce the virus stock used for animal inoculations. The virus stock had a titer of 10^6.5^ TCID_50_/ml on MDCK cells using the method of Reed and Muench [9].

### Administration of IFN-alpha and influenza A virus inoculation

Animals were administered Pegasys® (pegylated IFN-alpha 2a) intratracheally, intranasally, and intramuscularly with doses of 25 ug in 1 mL, 25 ug in 1 mL, and 50 ug in 0.5 mL, respectively. All dilutions were done in PBS. The intranasal/intratracheal/conjunctival influenza A virus inoculation procedureand the respiratory secretion sample collection procedure have been previously described [10]. Some animals were treated with IFN-alpha 24 hours before inoculation with 1 ml of the virus stock instilled into the trachea, 1 ml of virus stock dripped intranasally, and a drop of virus stock in each conjunctiva. Tracheal aspirate and blood samples were collected on days −8, −4, 0 (blood only), 1, 2, 3, 7, 14, and 28 (blood only) relative to the day of influenza A virus challenge.

### Titration of viral RNA in respiratory secretions

The concentration of virion-associated RNA (vRNA) in respiratory secretions was determined by RT-PCR as previously described [10].

### Tracheal sample cytokine mRNA expression levels

The method for assessment of host gene expression in tracheal secretions has been published [10]. Briefly, total RNA was isolated from the cellular pellets with TRIzol® (Invitrogen) according to manufacturer's instructions. RNA samples were DNase-treated and cDNA was prepared using random hexamer primers (Amersham-Pharmcia Biotech Inc.) and SuperScript III reverse transcriptase (Invitrogen). Cytokine mRNA levels were determined by RT-PCR as described previously [11], [12]. The GAPDH housekeeping gene and the target gene from each sample were run in parallel in the same plate. The reaction was carried out in a 96-well optical plate (Applied Biosystems) in a 25 µl reaction volume containing 5 µl cDNA plus 20 µl Mastermix (Applied Biosystems). All sequences were amplified using the 7900 default amplification program. The results were analyzed with the SDS 7900 system software, version 2.1 (Applied Biosystems). Cytokine mRNA expression levels were calculated from normalized ΔC_T_ values. C_T_ values correspond to the cycle number at which the fluorescence due to enrichment of the PCR product reaches significant levels above the background fluorescence (threshold). In this analysis, the C_T_ value for the housekeeping gene (GAPDH) is subtracted from the C_T_ value of the target (cytokine) gene (ΔC_T_). In general, the ΔC_T_ value for the influenza A-infected sample is then subtracted from the pre-infection ΔC_T_ value (ΔΔC_T_). Assuming that the target gene (cytokine) and the reference gene (GAPDH) are amplified with the same efficiency (data not shown), the increase in cytokine mRNA levels in test samples is then calculated as follows: increase = 2*^−ΔΔC^_T_* (user bulletin no. 2, ABI Prism 7700 Sequence Detection System: Applied Biosystems). Cytokine mRNA levels are expressed as the increase or decrease relative to the level for that cytokine in the individual monkey's pretreatment secretion sample. Because the mRNA expression level of housekeeping genes such as GAPDH can change under activating conditions, we were careful to use the same input amount of RNA for experimental samples in the PCR reactions. Regardless of the sample was collected the same input amount of RNA consistently resulted in similar PCR amplification (C_T_) values for GAPDH. Therefore, GAPDH expression in trachea was not differentially regulated among the animals in this study.

### Statistical Analysis

Statistics are reported as the mean and the standard error of the mean for each group using Prism 5.0a software (GraphPad) and data are presented as the probability and test used for analysis. Differences in vRNA levels, body temperature, and body weight in the treated and untreated animals after influenza virus inoculation were compared using a one-tailed t-test.

## Results

### Exogenous interferon administration induces antiviral immunity in the lungs of rhesus macaques

Based on previous experiments in rhesus macaques [13], six animals with body weights ranging from 7 to 10 kg were administered 100 ug of pegylated human recombinant IFN-alpha (Pegasys®, Roche Pharmaceuticals) locally and systemically. To induce the Type I IFN response in the respiratory tract, 25 ug of exogenous IFN-alpha in 1 mL PBS was administered drop wise intranasally and 25 ug IFN-alpha in 1 mL PBS was administered drop-wise intratracheally though a pediatric feeding tube. To induce the systemic Type I IFN responses, 50 ug of IFN-alpha in 0.5 mL of PBS was injected intramuscularly.

**Figure 1:**
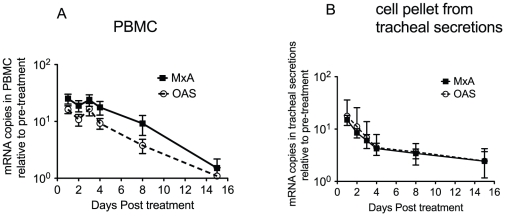
Innate antiviral responses induced in blood and lungs of rhesus macaques by exogenous IFN-alpha administration. Responses are represented as fold increase in specific gene mRNA levels as compared to pre-exposure mRNA levels isolated from an average of each animal's matched pre-exposure samples. *A.* Average MxA and OAS mRNA expression from PBMC (n = 6). *B.* Average MxA and OAS expression in the cell pellet from tracheal secretions (n = 6).

To determine the extent to which antiviral responses were induced by the IFN-alpha treatment, we monitored changes in the mRNA levels of two key IFN-stimulated genes, 2–5 oligoadenylate synthetase (OAS) and myxovirus resistance protein A (MxA). MxA, a GTPase protein encoded by the MX1 gene with potent antiviral activity, we chose to monitor MxA because it is a sensitive and specific biomarker of IFN bioactivity and it is used clinically to monitor the effect of IFN therapy [14]. The MxA and OAS mRNA levels of an animal after IFN-alpha administration are expressed as the increase relative to the average level in two pre-treatment samples from the same individual animal (Figure 1). IFN-alpha treatment resulted in increased OAS and MxA mRNA expression in PBMCs (Figure 1A) and tracheal lavages (Figure 1B) that peaked 24 hrs after the treatment.

### Exogenous IFN-alpha administration 24 hours prior to virus challenge decreases clinical signs of influenza A virus infection

**Figure 2:**
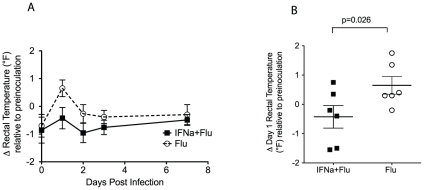
Effect of A/Memphis/7/01 inoculation on body temperature. *A.* Body temperature after influenza virus inoculation relative to the average of 3 temperatures readings collected before inoculation (mean +/− SE). B. Difference in body temperature 1 day after influenza virus inoculation relative to pre-inoculation. (P value for unpaired one-tailed t-test.).

**Figure 3:**
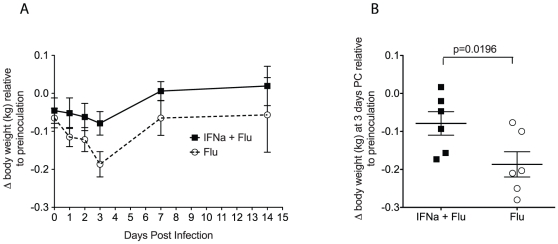
Effect of A/Memphis/7/01 inoculation on body weight. *A.* Body weight after influenza virus inoculation relative to the average of 3 weights measured before inoculation (mean +/− SE). B. Difference in body weight 3 days after influenza virus inoculation relative to pre-inoculation. (P value for unpaired one-tailed t-test.).

To determine the effect of IFN-alpha-induced immunity on the clinical signs of influenza A virus infection, body temperatures and weights before and after infection were monitored (Figures 2 and 3). Rectal temperatures were significantly lower in IFN-alpha-treated monkeys 24 hours after challenge with A/Memphis/7/01 compared to the control animals (p = 0.026, one-tailed T-test, Figure 2). In addition, by three days post infection, control animals had a 2.6 fold higher mean weight loss than the IFN-alpha treated animals (p = 0.039, one-tailed T-test, Figure 3).

### Exogenous IFN-alpha administration 24 hours prior to virus challenge blunts influenza A virus replication

**Figure 4:**
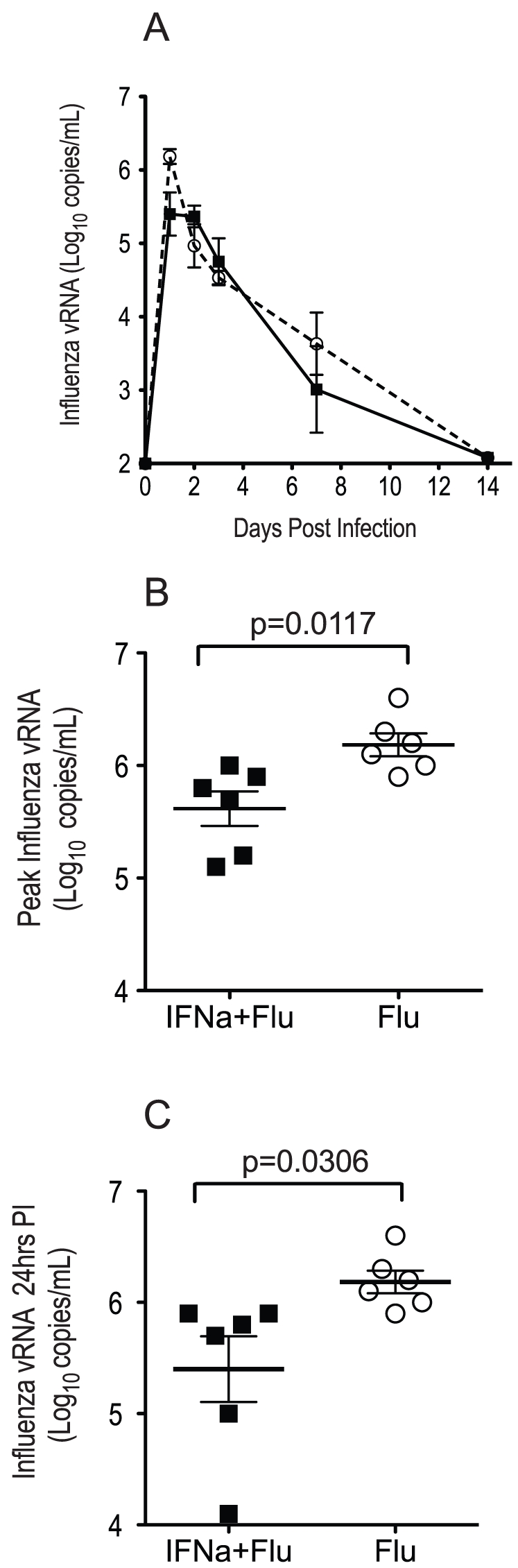
Effect of IFN-alpha administration 24 hours prior to virus challenge on A/Memphis/7/01 influenza virus replication in the lungs of rhesus macaques. A) Mean influenza A vRNA copy number (log_10_ copies/mL) from tracheal lavages of the IFN-alpha treated (solid line) and untreated animal (dashed line) groups over the period of observation. B) Comparison of peak vRNA levels in tracheal lavages after challenge. C) Comparison of 24-hour post-inoculation vRNA levels in tracheal lavages. (P values for unpaired one-tailed T-tests.).

To determine the effect of IFN-alpha-induced immunity on the influenza A virus replication vRNA levels in tracheal secretions were monitored (Figure 4). Influenza A virus RNA levels were significantly lower (p = 0.031) at 24 hrs PC (Figure 4) and peaked at a lower level (p = 0.012) in IFN-alpha treated animals compared to control animals (Figure 4).

## Discussion

The results of these experiments clearly demonstrate that systemic and respiratory mucosal administration of IFN-alpha induces expression of itself (data not shown) and interferon-stimulated genes in the trachea and blood of rhesus macaques. Further rhesus macaques treated with IFN-alpha 24 hours prior to influenza A virus challenge have about a 10-fold reduction in peak influenza RNA levels in tracheal secretions compared to the control animals. A log 10 reduction in influenza A virus RNA in tracheal secretions is the same level of virus suppression that prophylactic oseltamivir treatment can achieve in rhesus macaques challenged with A/Memphis/7/01 (H1N1) [10]. Prophylactic IFN-alpha reduces influenza virus replication as effectively as prophylactic osletamivir therapy. While the mechanism behind the effect of exogenous IFN-alpha was not determined, IFN-alpha administration rapidly induced ISG expression that peaked around 24 hours. By inoculating the RM with influenza virus at the peak of the ISG response virus replication was reduced for the first 24–48 hours of the infection. Thus the induction of innate antiviral immune responses is the most likely explanation for the observed effect of prophylactic IFN-alpha administration. It would be of considerable interest to determine if repeated IFN-alpha administration could produce more rapid clearance of the virus. Similarly ferrets treated prophylactically with recombinant ferret IFN-alpha control influenza replication in the nasal cavity [15]. Thus IFN-alpha induced innate antiviral effector mechanisms induced prior to infection can blunt seasonal influenza virus replication in ferrets and monkeys, and likely humans as well. However, it remains to be seen if IFN-alpha administered therapeutically after influenza virus infection can suppress virus replication.

Hundreds of effector genes are upregulated in response to virus infections, and the key antiviral effector molecule that controls replication varies with the specific virus [5]. Although it is unclear which of the ISGs are responsible for suppressing influenza A virus replication in rhesus macaques after IFN-alpha treatment, there is some indirect evidence that MxA may play a role. In contrast to the other ISGs, MxA mRNA expression is suppressed by influenza virus replication [10]. This result implies the virus is actively suppressing MxA expression, presumably to avoid the negative consequences it would have if expression of this gene was unrestrained. The mechanism by which MxA would control influenza virus replication in human cells is unknown. Mx1 the murine homologue of MxA is expressed in the nucleus and specifically blocks the replication of *Orthomyxoviruses* [16]. Because of its location, Mx1 directly inhibits primary transcription of influenza virus mRNA in the host cell nucleus [17]. In contrast, human MxA localizes to the smooth endoplasmic reticulum in the cytoplasm of human cells [18] and thus MxA would not be expected to affect primary transcription of influenza virus mRNA. When murine 3T3 cells line are engineered to constitutively express high levels of MxA and infected with influenza virus, viral mRNAs are transcribed normally by the virion-associated RNA polymerase in the nucleus. Viral protein synthesis and genome amplification is strongly inhibited in these cells although viral mRNAs direct viral protein synthesis in vitro and appeared to be efficiently transported to the cell cytoplasm. These results suggest that in mouse 3T3 cells MxA interferes with either intracytoplasmic transport of viral mRNAs, viral protein synthesis, or translocation of newly synthesized viral proteins to the cell nucleus [17], [19], [20]. More recent studies have shown that if MxA is artificially made to translocate into the nucleus of 3T3 cells, it binds newly synthesized influenza virus nucleoprotein preventing viral transcription [17], [19], [20]. MxA is likely to directly mediate the observed anti-influenza virus effects. Further study is needed to confirm that MxA is the key effector molecule that mediates the ability of IFN-alpha to decrease influenza virus replication in rhesus macaques and to determine if MxA expression can be therapeutically manipulated to treat influenza virus infections.
